# A Diketopiperazine, *Cyclo*-(L-Pro-L-Ile), Derived From *Bacillus thuringiensis* JCK-1233 Controls Pine Wilt Disease by Elicitation of Moderate Hypersensitive Reaction

**DOI:** 10.3389/fpls.2020.01023

**Published:** 2020-07-08

**Authors:** Ae Ran Park, Se-In Jeong, Hee Won Jeon, Jueun Kim, Namgyu Kim, Manh Tuan Ha, Mohamed Mannaa, Junheon Kim, Chul Won Lee, Byung Sun Min, Young-Su Seo, Jin-Cheol Kim

**Affiliations:** ^1^Department of Agricultural Chemistry, College of Agriculture and Life Sciences, Institute of Environmentally Friendly Agriculture, Chonnam National University, Gwangju, South Korea; ^2^Department of Chemistry, Chonnam National University, Gwangju, South Korea; ^3^Department of Integrated Biological Science, College of Natural Science, Pusan National University, Busan, South Korea; ^4^Drug Research and Development Center, College of Pharmacy, Daegu Catholic University, Gyeongbuk, South Korea; ^5^Forest Insect Pests and Diseases Division, National Institute of Forest Science, Seoul, South Korea

**Keywords:** pine wood nematodes, diketopiperazine, resistance-inducing bacteria, foliar application, moderate hypersensitive reaction, *cyclo*-(L-Pro-L-Ile), *Bacillus thuringiensis*

## Abstract

Pine wilt disease (PWD) caused by the pine wood nematode (PWN) *Bursaphelenchus xylophilus* is one of the devastating diseases affecting pine forests worldwide. Although effective control measurements are still missing, induction of resistance could represent a possible eco-friendly alternative. In this study, induced resistance-based *in vitro* and *in vivo* screening tests were carried out for selection of bacteria with the ability to suppress PWD. Out of 504 isolated bacteria, *Bacillus thuringiensis* JCK-1233 was selected for its ability to boost pathogenesis-related 1 (*PR1*) gene expression, a marker of systemic acquired resistance. Moreover, treatment of pine seedlings with *B. thuringiensis* JCK-1233 resulted in increased expression of other defense-related genes, and significantly inhibited PWD development under greenhouse conditions. However, *B. thuringiensis* JCK-1233 showed no direct nematicidal activity against *B. xylophilus*. To identify the effective compound responsible for the induction of resistance in *B. thuringiensis* JCK-1233, several diketopiperazines (DPKs) including *cyclo*-(D-Pro-L-Val), *cyclo*-(L-Pro-L-Ile), *cyclo*-(L-Pro-L-Phe), and *cyclo*-(L-Leu-L-Val) were isolated and tested. Foliar treatment of pine seedlings with *Cyclo*-(L-Pro-L-Ile) resulted in suppression of PWD severity and increased the expression of defense-related genes similarly to *B. thuringiensis* JCK-1233 treatment. Interestingly, treatment with *B. thuringiensis* JCK-1233 or *cyclo*-(L-Pro-L-Ile) showed moderately enhanced expression of *PR-1*, *PR-2*, *PR-3*, *PR-4*, *PR-5*, and *PR-9* genes following inoculation with PWN compared to that in the untreated control, indicating that they mitigated the burst of hypersensitive reaction in susceptible pine seedlings. In contrast, they significantly increased the expression levels of *PR-6* and *PR-10* before PWN inoculation. In conclusion, foliar spraying with either *B. thuringiensis* JCK-1233 culture suspension or DPKs could induce resistance in pine seedlings, thereby alleviating the serious damage by PWD. Taken together, this study supports aerial spraying with eco-friendly biotic or abiotic agents as a valuable strategy that may mark an epoch for the control of PWD in pine forests.

## Introduction

Pine wilt disease (PWD) caused by the pine wood nematode (PWN) *Bursaphelenchus xylophilus* is one of the most destructive diseases damaging pine forests. The transmission of PWN occurs *via* pine sawyer beetles (*Monochamus* spp.), which are attracted to pine trees for feeding or oviposition (Mamiya and Enda, 1972). Since the first incidence of PWD was reported in 1905 in Nagasaki, Japan (Yano, 1913), PWD has spread quickly throughout East Asia, Europe, and even North America, threatening pine forests worldwide (Yi et al., 1989; Mota et al., 1999).

Despite the advances in the study of PWD, effective control measures have not yet been developed. As the habitat of pine trees is very wide areas and, in many cases, poorly accessible, such as cliffs and steep mountains, operations by manual labor are impractical. In addition, most pine trees infected by PWN are killed rapidly, as PWN is an endoparasite that is very difficult to control. Consequentially, PWD has caused enormous economic losses with environmental impacts worldwide and thus, is considered a serious threat to be dealt with (Tóth, 2011).

Current PWD control methods depend mainly on the removal of infection sources or other preventative measures, such as fumigation, burning, clear-cutting, breeding, aerial insecticide spraying, and trunk injection (Takai et al., 2003; Kwon et al., 2005; Nose and Shiraishi, 2008; Bonifácio et al., 2014). However, traditional chemical control using chemical compounds, including methyl bromide and phosphine, is known to cause severe environmental problems because of the high toxicity and potential to induce resistance among parasitic nematodes (Bell, 2000). Recently, with the growing public interest in eco-friendly control methods, biological control agents of plant-parasitic nematodes have received greater attention as an environmentally safe alternative for plant protection. Specifically, agricultural application of plant-associated bacteria, originated from phyllosphere or rhizosphere, exhibited the ability to reduce the incidence or severity of soil-borne diseases (Vallad and Goodman, 2004). Induced resistance is among the reported biocontrol mechanisms for management of diseases that do not exert a direct selective pressure on the pathogen population. Specifically, systemic acquired resistance (SAR) is activated throughout higher plants after being exposed to elicitors from virulent, avirulent, or nonpathogenic microbes, or chemical stimuli such as salicylic acid (SA), which then confers long-lasting protection against a broad spectrum of phytopathogenic microorganisms (Vallad and Goodman, 2004).

Studies on plant immunity have indicated that endophytic bacteria increase plant resistance to pathogens through signaling crosstalk in various plants. However, few studies have addressed the use of bacteria-mediated induction of resistance for PWD management. Previously, inoculation with avirulent *B. xylophilus* was shown to induce resistance against PWD in pine trees, suggesting that the mechanism of induced resistance in pine trees has the potential for biological control against PWD (Kosaka et al., 2001). Interestingly, foliar sprays of SAR inducers in pineapple reduced the reproduction of plant-parasitic nematodes such as *Meloidogyne javanica* and *Rotylenchulus reniformis*, which damage the pineapple root system (Rohrbach and Apt, 1986; Chinnasri et al., 2006). Some rhizobacteria also elicit systemic resistance that may be dependent on SA (Kloepper and Ryu, 2006). It was reported that even though susceptible pine trees are infected with virulent PWNs by vector beetles feeding, the ability of pine trees to activate defensive responses to the infection may reduce the nematode migration and proliferation rates within the plant tissues to some extent (Kuroda, 2008). Therefore, we predicted that the induction of resistance by foliar application with biological agents, such as endophytic bacteria, could suppress the dispersal of PWN and limit the serious damage caused by PWD.

The induced resistance in plants is divided into systemic acquired resistance (SAR) and induced systemic resistance (ISR) (Van Loon et al., 1998). Although SAR is induced by a prior pathogen infection at a local tissue, it can protect the rest of the plant from a second infection. ISR is elicited by plant growth-promoting rhizobacteria (PGPR) and confers protection of plants to a broad spectrum of attackers. SAR is associated with the SA signaling pathway, whereas ISR is mediated by the JA and ET signaling pathways. Although it has been known that SAR and ISR are clearly different, recent studies have been reported that they are interconnected by crosstalk of SA, ET, and jasmonic acid signaling from some rhizospheric *Bacillus* strains (Niu et al., 2016; Song et al., 2017). For example, PGPR *Bacillus cereus* AR156 installs ISR and enhances SAR with increased PR-1 protein expression in plants (Niu et al., 2016). Pathogenesis-related (PR) genes are widely expressed downstream the SA, jasmonic acid (JA), and ethylene (ET) pathways in plants, which play important roles in the inducible defense mechanism in plants against pathogens, facilitating plant adaptation to the environment (Hoffmann-Sommergruber, 2002). Specifically, the *PR-1* gene is used as a marker for the SA-dependent signal transduction pathway and for the study of defense gene expression in plants (Ono et al., 2004). Therefore, transgenic *Arabidopsis* plants transformed with the *PR-1*-promoter fused to the *β-glucuronidase* (*GUS*) or *luciferase* have been used as a model system for high-throughput screening of bacterial activators that enhance disease-resistance mechanisms in various plants (Ogura et al., 2005; Narusaka et al., 2006). Therefore, we predicted that endophytic bacteria may stimulate the expression of the *PR-1* gene and influence their resistance-inducing activities.

In this study, endophytic bacteria isolated from several sources were screened for their possible induction of resistance against PWD, using a sequence of *Arabidopsis* plants with the *PR-1*-promoter fused to *GUS*, *in vitro* pine callus, and *in vivo* pine seedling assay systems. The objectives of this study were to select resistance-inducing bacteria capable of managing PWD by foliar application, identify the bioactive compounds responsible for the induction of resistance, and characterize the functional mechanism employed in pine trees by the selected bacteria and their bioactive compounds.

## Materials and Methods

### Nematodes, Plant Materials, and Callus Culture

The pine wood nematode (PWN) *B. xylophilus* was isolated from infected pine trees and provided by the National Institute of Forest Science (NIFoS; Seoul, South Korea). Initially, PWN was cultured on the mycelia of *Botrytis cinerea* fully grown on potato dextrose agar (PDA, Difco; Becton, Dickinson and Company, MD, USA) at 25°С for propagation (Maehara and Futai, 2000). After 7 days of incubation, the propagated nematode was harvested using the funnel technique (Baermann, 1917), rinsed three times with sterilized distilled water and then prepared as an aqueous suspension of *B. xylophilus* for subsequent experiments.

Seeds of the *Arabidopsis* plant (*Arabidopsis thaliana* ecotype Columbia (Col-0)) genetically engineered with the *GUS* reporter gene fused to the *PR-1* promoter were provided by Y. C. Kim (College of Agriculture and Life Science, Chonnam National University, South Korea). The seeds were surface sterilized in 5% sodium hypochlorite followed by immersion for 3 min in 70% ethanol. After washing with sterile distilled water, they were left to imbibe in sterile water containing 0.1% agarose (Gibco; Thermo Fisher Scientific INc., MA, USA) in the dark at 4 for 3 days. Subsequently, the seeds were allowed to germinate on plates containing 1× Murashige and Skoog (MS) salt mixture and 0.5 g/L 2-(*N*-morpholino) ethanesulfonic acid (MES), pH 5.8, in 1% Duchefa agar (Duchefa Biochemie, Haarlem, The Netherlands). Seedlings were grown in a growth chamber (VS-3DM-600; Hanbaek, Bucheon, South Korea) under photoperiodic cycles of 16-hour light/8-hour dark at 22 with 70% humidity.

For the *in vivo* pathogenicity assay, three- or four-year old red pine (*Pinus densiflora*) and black pine (*P. thunbergii*) saplings with average height of 40 cm and average diameter of 0.5 cm were obtained from Daelim Farm (Okcheon, South Korea) and then transplanted to 15-cm diameter pots containing sterilized nursery soil in the greenhouse, keeping an average temperature of 25°C.

For the *in vitro* assay, *Pinus* calli were obtained from NIFoS (Seoul, South Korea). Calli were taken aseptically from embryos of *P. densiflora* and cultured in Litvay medium (LM; Thomas Scientific Inc., NJ, USA), including vitamins with 2 μg/ml 2,4-chlorophenoxyacetic acid and 1 μg/ml 6-benzyl-aminopurine solution at 24 under dark conditions.

### Isolation and Incubation of Endophytic Bacteria

Endophytic strains were isolated from agronomic plants and grove trees of five regions in South Korea (Daejeon, Gwangju, Jeongeup, Busan, and Sacheon). The agronomic plants tested were tomato, pepper, and onion. The grove trees used in this study included cherry and peach trees. Individual leave, stem, and root samples were put into plastic bags, placed in a cool box for transportation, and stored at 4°С. Plant samples were surface sterilized for 10 s with 2% sodium hypochlorite and rinsed five times in sterile distilled water. Sterilized plant samples were dissected into 1-cm pieces and macerated with a sterile mortar and pestle. Each 1 g plant sample was suspended in 10 ml of sterile distilled water and shaken vigorously for 2 min. The supernatant was serially diluted in sterile distilled water (10^−1^ to 10^−7^), and plated on tryptic soy agar medium (TSA, Difco, MD, USA). After incubation at 30°С for 1–2 days, each strain was streaked on TSA and then a single colony was isolated. Isolated bacterial strains were stored cryogenically in 20% glycerol at −70°С. For *in vitro* and *in vivo* bioassays, bacterial strains were inoculated in tryptic soy broth (TSB, Difco, MD, USA) for 3 days at 30°С with agitation (200 rpm).

### Histochemical Staining for GUS Activity in Arabidopsis Leaves

Four-week-old *Arabidopsis* seedlings from the *PR-1pro::GUS* line were used to assess the resistance inducting activity of endophytic bacteria, which were isolated from several plants and their rhizospheric soils. For GUS staining of *Arabidopsis* leaves, leaf discs (5 mm diameter) were placed in 96 well plates containing the culture filtrate of bacterial strains and then the plates were incubated for 12 h at 22 with relative humidity above 70% under light conditions. After treatment, GUS activity was measured as described by Jefferson et al. (1987). Prior to the staining reactions, the treated leaves were fixed in a fixation solution (0.3% formaldehyde, 10 mM MES, pH 5.6, and 0.3 M mannitol) for 1 h on ice. The staining reaction was performed in 50 mM sodium phosphate buffer (pH 7.0) that contained 10 mg/ml 5-bromo-4-chloro-3-indolyl-β-_D_-glucuronic acid (X-Gluc) and 0.02% (w/v) Triton X-100 for 24 h at 22 in the dark. After staining, leaf discs were decolorized in 7% (v/v) ethanol for 24 h and rinsed with water. Each experiment was run in triplicates.

### In Vitro Screening of Bacterial Strains That Induce PR-1 Gene in Pinus callus

The endophytic bacteria that were selected based on GUS activity in *PR-1pro::GUS* Arabidopsis line were used for the *in vitro* assay that analyzed their *PR-1* gene expression inducing activity in *Pinus* callus. Selected endophytic bacteria were cultured up to an OD_600_ = 0.8 at 30 in TSB for the *in vitro* assay. A bacterial suspension (500 μl) was treated with *P. densiflora* callus (100 mg) and then incubated at 24 using a digital rocker at 50 rpm under dark conditions. The untreated controls for the *in vitro* experiments were performed applying the same amount of sterile TSB. Each experiment was run in triplicates.

After incubation with the bacterial suspension for 1 day, total *P. densiflora* callus RNA was extracted using CTAB extraction buffer with elimination of high viscosity and excessive polysaccharides (Azevedo et al., 2003). Then, total RNA was further purified using RNeasy mini kit (Qiagen, Valencia, CA, USA), according to the manufacturer’s recommendations. cDNA libraries were prepared from total callus RNA with oligo (dT) primers and SuperScript™ IV reverse transcriptase (Invitrogen Inc., Carlsbad, CA, USA), according to the manufacturer’s protocols. The PCR primers of the *PR-1* gene used in this study (**Table 1**) were synthesized by Genotech (Daejeon, Korea).

**Table 1:** Primers used in this study.

Gene	Sequence (5’→3’)	Reference
PR-1 For	TGCCCCTTCAGGTAAATCGT	Hirao et al. (2012)
PR-1 Rev	GCGGGTCGTAGTTGCAGATAA	
PR-2 For	CGACAACATTCGCCCCTTCT	
PR-2 Rev	CTGCAGCGCGGTTTGAATAT	
PR-3 For	CCATCGAAGCCCAGGTAATTT	
PR-3 Rev	AGCCGGGAAGCAATATTATGGT	
PR-4 For	CCCCGTTACTGTCAATTGCAT	
PR-4 Rev	AAAGCGTGACGGTGCGTATT	
PR-5 For	GAACCAGTGCCCATACACAGTCT	
PR-5 Rev	CCTGCGGCAACGTTAAAAGTC	
PR-6 For	TGCTGGCGGCATCTATTTTA	
PR-6 Rev	TAACACCTGCGCAAATGCA	
PR-9 For	ACACCACCGTGCTGGACATT	
PR-9 Rev	GTGCGGGAGTCGGTGTAGAG	
PR-10 For	TGTCTCAAGTGGAGGCAAGGA	
PR-10 Rev	AAGCGACAATTTCAGGCAAAAC	
EF-1α For	GGGAAGCCACCCAAAGTTTT	
EF-1α Rev	TACATGGGAAGACGCCGAAT	
PdPR-4 For	TGTGACGAATCCTTCAACGC	Lee et al. (2019)
PdPR-4 Rev	AAAGCCGCGGTTTCAAGATC	
PdCHI For	TTCATCACAGCTGCCAATGC	
PdCHI Rev	ATGCTCCAGTTTCGTGCATC	
PdBGL2 For	AAGTCCGTGCATTCTCAACG	
PdBGL2 Rev	TCCGCCATGGAAAATTTGGG	

Determination of relative mRNA expression was carried out in a real-time PCR detection system (Bio-Rad CFX 96; Bio-Rad Laboratories, Hercules, CA, USA). cDNA was analyzed using iQ™ SYBR Green supermix (Bio-Rad Laboratories) in a 20 μl volume. Data were analyzed using BioRad CFX Manager Version 2.1. Relative fold changes in mRNA between treatments were determined based on the ΔΔCT method after normalizing to the housekeeping gene elongation factor 1α (EF-1α) (Livak and Schmittgen, 2001). Samples were run in triplicate and averaged.

### Efficacy of JCK-1233 in the Control of PWD by B. xylophilus on Pinus densiflora and P. thunbergii Seedlings

The disease control efficacy of the JCK-1233 bacterial strain was evaluated against PWD on three- and four-year-old *P. densiflora* (black pine) and *P. thunbergii* (red pine) seedlings with an average height of 40 cm and an average root-collar calliper of 0.5 cm. JCK-1233 were cultured in TSB at 30°C for 24 h with shaking at 150 rpm. Each culture was diluted using distilled water containing Tween 20 (250 mg/l) to a final concentration of 8 × 10^8^ colony-forming units (cfu)/ml using a UV–VIS spectrophotometer (UV-1601; Shimadzu Co., Kyoto, Japan). Black pine and red pine seedlings pre-treated with Tween 20 (5 ml, 250 mg/l) per seedling were foliar sprayed twice with a JCK-1233 bacterial suspension (5 ml/seedling) at one-week interval. Distilled water containing Tween 20 (250 mg/l) was used as an untreated control. Emamectin benzoate (20 mg/ml) was supplied from Syngenta Korea (Seoul, South Korea) and used once as a positive control for treatment by trunk injection (100 μl/seedling). After one week from trunk injection with emamectin benzoate or the second treatment with the bacterial suspension, pine seedlings were inoculated with PWN as previously reported by Kwon et al. (2010). After making a small slit with a surface-sterilized knife in the stem of the seedlings, a small piece of absorbent cotton was inserted into the slit, and a water suspension of nematodes (2,000 nematodes/100 μl) was pipetted onto the absorbent cotton. The slits were then covered with Parafilm to prevent drying. PWD severity was evaluated according to the wilting and consequent discoloration area of the needles (Proença et al., 2010). The experiments were repeated twice in five replicates.

### In Vitro Nematicidal Activity of JCK-1233 Culture Filtrates Against B. xylophilus

The nematicidal activity of JCK-1233 culture filtrates was evaluated testing their effect on the mortality of PWN *B. xylophilus*. Treatments were performed in 96 well tissue culture plates containing approximately 50 PWNs/well. To prevent solution evaporation, the plates were covered and kept in the dark at 25°C with gentle shaking. Three days after exposure, the PWNs were moved to tap water and grouped into motile and immotile categories based on observations made under a light microscope (Leica DM IL LED; Leica Microsystems CMS GmbH, Wetzlar, Germany) after pricking their bodies with a fine needle. PWNs that did not move and retained a stiff and straight body shape even after pricking with a needle were considered dead. TSB medium was used as a negative control. The experiment was repeated twice with triplicate. To analyze the nematicidal activity of JCK-1233 against PWNs, the mortality of PWNs was converted to percentage mortality and corrected using the formula of Schneider-Orelli (1947): Mortality (%) = [(mortality percentage in treatment − mortality percentage in the negative control)/(100 − mortality percentage in the negative control)] × 100. The nematicidal activities of JCK-1233 were evaluated analyzing the mortality of PWNs over a concentration range of 0.63 to 20%. The experiments were repeated twice in triplicate.

### Molecular Identification of JCK-1233

A JCK-1233 isolate showing induced resistance activity in pine seedlings was identified by *recA* nucleotide sequence analysis. The genomic DNA of the JCK-1233 isolate was prepared using a DNeasy Blood and Tissue kit (Qiagen, Hilden, Germany) following the manufacturer’s recommendations. PCR amplification of the *recA* gene was performed using the universal bacterial primer pair recA-F (5′-GATCGTCARGCAGSCYTWGAT-3′)/recA-R (5′-TTWCCRACCATAACSCCRAC-3′) in a 20 μl reaction mixture containing genomic DNA (2 μl), primers (1 μl of each, 10 pM), sterilized distilled water (16 μl), and Accupower^®^ PCR premix (1 μl) (Bioneer Corp., Daejeon, South Korea). The PCR conditions were 95 for 10 min, followed by 35 cycles of 95 for 30 s, 49 for 30 s, and 72 for 1 min, and then a final extension at 72 for 5 min. The result from the *recA* sequencing was used to identify JCK-1233 based on the National Center for Biotechnology Information (NCBI) blast database. Sequence alignment and phylogenetic analysis were performed using the neighbor-joining (NJ) method with MEGA 6, with the number of bootstrap trials set to 1000. The Kimura 2-parameter model was selected as the best model to construct the tree for NJ (Tamura et al., 2013).

### Extraction and Isolation of Potential Resistance Inducers From B. thuringiensis JCK-1233 Strain Cultures

To find the chemicals giving inducible resistance to plants from the culture filtrates of *B. thuringiensis* JCK-1233, the strain was pre-cultured in in tryptic soy broth (TSB) medium overnight at 37 . Then, JCK-1233 was grown in TSB medium to an OD_600_ of 0.8. The cultured broth of TSB-1233 (72 L) was condensed to 10 L on a rotary evaporator *in vacuo* at 40°C. Then, the condensed broth was partitioned with CH_2_Cl_2_ to yield different fractions. The CH_2_Cl_2_ soluble fraction was subjected to silica gel column chromatography (CC) and eluted with CH_2_Cl_2_-MeOH (100:0 to 0:100, gradient, v/v), producing 13 fractions (C1–C13). Fraction C7 was further fractionated by silica gel CC and eluted with CH_2_Cl_2_-acetone (20:1, v/v) to give eight sub-fractions (C7.1–C7.8). Sub-fraction C7.5 was purified by semi-preparative RP-HPLC [Gilson Trilution System, Middleton, WI, USA; YMC Pak ODS-A column (20 × 250 mm, 5 μm particle size), YMC Co., Kyoto, Japan; UV detection at 210 nm] using MeOH and H_2_O in a 0.1% TFA gradient (40:60–70:30, v/v) at a ﬂow rate of 5 ml/min as a mobile phase. Fraction C11 was further fractionated by silica gel CC and eluted with CH_2_Cl_2_-acetone (10:1, v/v) to produce seven sub-fractions (C11.1–C11.7). Following a similar procedure to that used for C7.5, sub-fraction C11.4 was subjected to semi-preparative RP-HPLC using MeOH and H_2_O in a 0.1% TFA gradient (50:50–70:30, v/v) at a ﬂow rate of 5 ml/min as a mobile phase.

### Characterization of Potential Resistance Inducers Isolated From B. thuringiensis JCK-1233 Strain Cultures

The optical rotations were measured using a Jasco P-1020 polarimeter (JASCO, Tokyo, Japan). The electrospray ionization (ESI) mass spectra were performed on an AGILENT 1100 LC-MSD trap spectrometer (Agilent Technologies, Palo Alto, CA, USA). High-resolution electrospray ionization mass spectra (HR-ESI-MS) were obtained from an Agilent 6530 Accurate-Mass Q-TOF LC/MS system (Agilent technology, Santa Clara, CA, USA). NMR spectra were recorded with a Bruker 500 MHz spectrometer (Bruker, Karlsruhe, Germany) using tetramethylsilane (TMS) as the internal standard. Silica gel (Merck, Darmstadt, Germany; 63−200 μm particle size) and RP-18 (Merck, 75 μm particle size) were used for CC. TLC was performed using Merck silica gel 60 F_254_ and RP-18 F_254_ plates. Preparative reversed-phase (RP)-HPLC was performed using a Gilson Trilution System with an UV detector (UV/VIS-156) and a YMC Pak ODS-A column (20 × 250 mm, 5 μm particle size, YMC Co., Kyoto, Japan). HPLC solvents were purchased from Burdick & Jackson, USA.

### Effect of Foliar Spray and Trunk Injection of DPKs Produced by B. thuringiensis JCK-1233 Against PWD

The disease control efficacy of diketopiperazines (DPKs) produced by JCK-1233 was evaluated against PWD on three- and four-year-old *P. thunbergii* (black pine) seedlings. Four DPKs isolated from JCK-1233 culture broth were diluted using distilled water containing Tween 20 (250 mg/l) to a working concentration of 1 mM and then used for trunk injection and foliar spray treatments. For trunk injection, four DPKs (1 mM) and emamectin benzoate (20 mg/ml) containing 5% MeOH were treated with 100 μl per seedling. For foliar application, four DPKs (1 mM, 5 ml per seedling) and JCK-1233 culture (OD_600_ = 0.8, 5 ml per seedling) were foliar sprayed on Tween 20 pre-treated seedlings twice at one-week interval. For untreated controls, the same amount of sterile TSB in distilled water containing Tween 20 (250 mg/l) for foliar spray and 5% MeOH for trunk injection was applied. After one week from the second foliar spray and trunk injection treatments, pine seedlings were inoculated with PWN (2,000 nematodes/100 μl). PWD severity was evaluated according to the wilting area of the seedling. The experiments were repeated twice in five replicates.

### Effect of B. thuringiensis JCK-1233 and cyclo-(L-Pro-L-Ile) on the Expression of Defense Related Genes In Vivo

*P. thunbergii* (black pine) was used to analyze the effect on the defense related genes expression in pines. JCK-1233 was cultured to OD_600_ = 0.8 at 30 in TSB and then JCK-1233 bacterial suspension containing Tween 20 (250 mg/l, 5 ml per seedling) used for foliar spray. The selected diketopiperazine, *cyclo*-(L-Pro-L-Ile), was diluted using distilled water containing Tween 20 (250 mg/l) to a working concentration of 1 mM and then used for foliar spray (5 ml per Tween 20 pre-treated seedling twice at one-week interval). For untreated controls, the same amount of sterile TSB in distilled water containing Tween 20 (250 mg/l) was applied. After one week from the second treatment, pine seedlings were inoculated with PWN (2,000 nematodes/100 μl). Three replicates were performed for each treatment.

At 1 day after the first treatment (1 DAT), 1 day after the second treatment/8 days after the first treatment (8 DAT), and 1 day and 3 days after inoculation with PWN (1 DAI and 3 DAI), *P. thunbergii* total RNA was extracted from the pine needles using CTAB extraction buffer with elimination of high viscosity and excessive polysaccharides (Azevedo et al., 2003). Then, total RNA was further purified using IQeasy™ plus plant RNA extraction mini kit (iNtRON, Seongnam, South Korea), according to the manufacturer’s recommendations. cDNA libraries were prepared from total pine needle RNA using oligo (dT) primers and SuperScript™ IV reverse transcriptase (Invitrogen Inc., Carlsbad, CA, USA), according to the manufacturer’s protocols. The PCR primers used in this study (**Table 1**) were synthesized by Genotech (Daejeon, Korea).

Determination of relative mRNA expression was carried out in a real-time PCR detection system (Bio-Rad CFX 96; Bio-Rad Laboratories, Hercules, CA, USA). cDNA was analyzed using iQ™ SYBR Green supermix (Bio-Rad Laboratories) in a 20 μl volume. The data were analyzed using BioRad CFX Manager Version 2.1. Relative fold changes in mRNA between treatments were determined based on the ΔΔCT method after normalizing to the housekeeping gene elongation factor 1 alpha (Livak and Schmittgen, 2001). The samples were run in triplicate and averaged.

### Statistical Analysis

The parameters measured in this study were designed to evaluate the efficacy of JCK-1233 and DPKs against PWN. The analyses were conducted separately for *in vitro* and *in vivo* experiments. All data were analyzed for homogeneity of variance using the SPSS statistical analysis software (version 21.0 for Windows; SPSS, Chicago, IL, USA). The data were expressed as means ± standard error of replicates and evaluated by one-way analysis of variance (ANOVA). Statistical differences among treatments were determined according to Duncan’s multiple-range test (*p <* 0.05).

## Results

### Primary Screening of Bacteria-Induced Resistance in Arabidopsis

Five hundred and four bacterial strains were isolated from plants of five different regions in Korea. The isolated bacteria were screened for their potential resistance-inducing abilities using transgenic *Arabidopsis* plant lines containing the β-glucuronidase (*GUS*) construct fused to the *PR-1* promoter, which are known to visualize the ability to elicit the SA signaling pathway when exposed to potential resistance inducers. After co-incubation of bacterial cultures with leaf discs from the *PR-1pro::GUS Arabidopsis* line, 24 isolates out of 504 endophytic bacteria showed increased transcriptional GUS activity compared to that of the untreated control (**Supplementary Data S1**).

### Effect of the Selected Bacteria on PR-1 Transcript Expression in Pinus Calli

Among the 24 selected bacterial strains from the previous *PR-1pro::GUS Arabidopsis* assay, only 8 strains were shown in the pine callus assay to increase the expression of *PR-1* at least 1.3-fold compared to that in untreated controls (**Table 2**). Specifically, bacterial strain JCK-1233-treated calli showed the highest increase in *PR-1* gene expression (3.59-fold compared to that in the untreated control). Based on these results, JCK-1233 was selected for further experiments as a potent candidate for the induction of resistance in pine trees.

**Table 2:** Relative transcription level of the SA marker *PR1* in *Pinus densiflora* calli inoculated with the initially selected endophytic bacterial strains.

Strain	*PR1*	Strain	*PR1*
JCK-757	2.19 ± 0.16	JCK-1229	1.08 ± 0.42
JCK-758-1	0.94 ± 0.41	JCK-1233	3.59 ± 0.88
JCK-758-2	0.58 ± 0.12	JCK-1266	0.73 ± 0.19
JCK-761	1.30 ± 0.14	JCK-1287	0.66 ± 0.09
JCK-767	0.24 ± 0.10	JCK-1288	1.49 ± 0.34
JCK-947	0.82 ± 0.13	JCK-1307	0.42 ± 0.16
JCK-1005	0.67 ± 0.09	JCK-1308	0.64 ± 0.21
JCK-1180	2.04 ± 0.49	JCK-1309	1.31 ± 0.42
JCK-1182	0.55 ± 0.12	JCK-1318	1.89 ± 0.33
JCK-1187	2.25 ± 0.68	JCK-1320	0.94 ± 0.34
JCK-1217	0.75 ± 0.11	JCK-1328	1.09 ± 0.15
JCK-1222	0.12 ± 0.02	JCK-1333	0.85 ± 0.31

Data was presented as the mean ± standard deviation of three biological replicates.

### Efficacy of JCK-1233 in the Control of PWD by B. xylophilus on P. densiflora and P. thunbergii Seedlings

**Figure 1:**
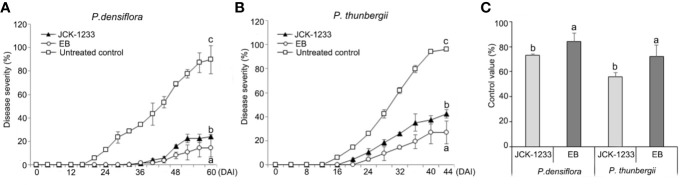
Effect of the JCK-1233 culture broth against pine wilt disease caused by pine wood nematodes in pine seedlings. Disease severity after inoculation with pine wood nematodes in **(A)** red pine (*Pinus densiflora*) seedlings, and **(B)** black pine (*Pinus thunbergii*) seedlings. **(C)** Disease control efficacy at 28 days after inoculation (DAI) in *P. densiflora* and *P. thunbergii* seedlings. Data was represented as the mean and standard error of two runs with five replicates per run. Different lower case letters shown values that are significantly different (*p <* 0.05) level by Duncan’s test.

Treatment with a JCK-1233 culture suspension significantly reduced PWD severity in nematode-inoculated *P. densiflora* and *P. thunbergii* seedlings (**Figure 1**). Disease severity in *P. densiflora* seedlings treated by foliar spray with a JCK-1233 culture suspension was significantly reduced compared to the control (24.2% compared to 89.8% in treated and control samples, respectively). The control efficacy of JCK-1233 treatment was comparable to that of EB-treated seedlings, in which disease severity was 14.5% (**Figure 1A**). Moreover, wilting in EB and JCK-1233-treated *P. densiflora* seedlings appeared gradually, starting from 36 DAI with PWNs, while wilting in untreated controls of *P. densiflora* seedlings advanced rapidly starting from 20 DAI. Although the control efficacy of EB treatment (83.9%) was slightly higher than that of JCK-1233 treatment, JCK-1233 treatment showed a significant control efficacy of 73.1% against PWD in *P. densiflora* seedlings (**Figure 1C**).

A similar control efficacy of JCK-1233 treatment was observed in *P. thunbergii* seedlings, in which disease severity reached 42.5%, compared to 96.3% in untreated controls and 26.7% in EB treated seedlings (**Figure 1B**). In addition, wilting in untreated *P. thunbergii* appeared earlier (16 DAI) than in EB- and JCK-1233-treated-*P. thunbergii* seedlings, in which wilting appeared gradually from 24 and 20 DAI, respectively. In agreement with the results on *P. densiflora* seedlings, JCK-1233 treatment also showed a significant control efficacy against PWD in *P. thunbergii* seedlings, although its control efficacy (55.8%) was a little lower than that of EB treatment (72.3%) (**Figure 1C**).

### In Vitro Nematicidal Activity of JCK-1233 Culture Suspensions Against B. xylophilus

The effect of JCK-1233 culture suspensions on *B. xylophilus* juvenile mortality was determined at 3 days after exposure. There was no effect of JCK-1233 treatment on *B. xylophilus* juvenile mortality at the tested concentrations (0.63–20%) compared to the TSB control treatment (0.8–1.9 and 2.3%, respectively), whereas treatment with EB exhibited more than 99% mortality from a concentration of 0.33 μg/ml (**Table 3**). Therefore, JCK-1233 does not seem to have a direct nematicidal activity against *B. xylophilus*.

**Table 3:** The *in vitro* nematicidal activity of JCK-1233 culture filtrates.

Sample	Concentration	Mortality (%)	STD
Emamectin Benzoate (μg/ml)	3	100.0a	0.0
	1	99.0a	0.9
	0.33	98.9a	2.0
	0.11	64.0b	2.8
	0.04	51.3c	5.3
	0.01	17.2d	1.5
JCK-1233 (%)	20	1.9e	2.0
	10	1.9e	1.8
	5	0.8e	1.3
	2.5	0.7e	1.3
	1.25	2.2e	0.1
	0.63	0.8e	1.3
TSB	–	2.3e	0.8

Each value represents the mean and standard deviation of two runs with three replicates per run. Different lower case letters shown values that are significantly different (p < 0.05) level by Duncan’s test.

### Molecular Identification of JCK-1233

**Figure 2:**
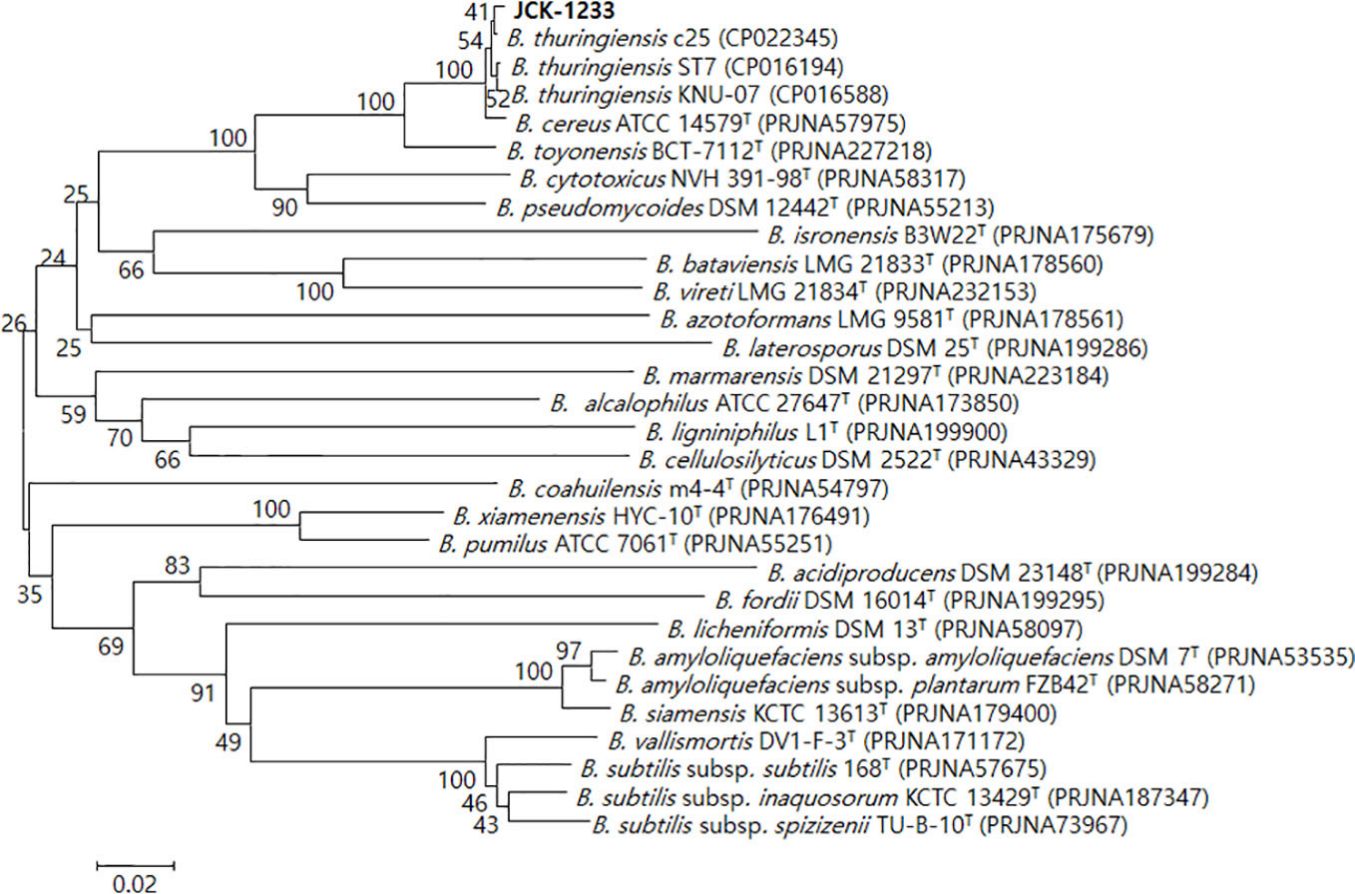
Phylogenetic tree derived from a distance analysis of *recA* gene sequences in JCK-1233. The sequences were aligned using MEGA 6.0 software. The phylogenetic tree was constructed using the neighbor-joining (NJ) method with bootstrap analysis (1,000 trials). The selected strain was identified as *Bacillus thuringiensis*, which was separated in an exclusive cluster. Bars indicate the percentage of sequence divergence. T represents the type strains.

The selected bacterial isolate JCK-1233 was identified as *B. thuringiensis* based on BLAST and phylogenetic analyses of the amplified *recA* gene sequence (**Figure 2**). The amplified genes were registered in GenBank under the accession number MT024187. *B. thuringiensis* JCK-1233 was deposited in the KCCM (Korean Culture Center of Microorganisms, Seoul, Korea) as KCCM 14085BP.

### Extraction and Isolation of Putative Active Compounds From B. thuringiensis JCK-1233 Strain Culture

The condensed broth of JCK-1233 (10 L) was partitioned with CH_2_Cl_2_, yielding CH_2_Cl_2_ (2.3 g) fractions. Using silica gel column chromatography (CC), the CH_2_Cl_2_ soluble fraction (2.3 g) was divided into 13 fractions (C1–C13). Fraction C7 (510 mg) was further fractionated by silica gel CC and eluted with CH_2_Cl_2_-acetone (20:1, v/v) to give eight sub-fractions (C7.1–C7.8). Sub-fraction C7.5 (253.5 mg) was purified by semi-preparative RP-HPLC using MeOH and H_2_O in a 0.1% TFA gradient (40:60–70:30, v/v) at a ﬂow rate of 5 ml/min as a mobile phase to give compounds 2 (40 mg) and 3 (55 mg). Fraction C11 (255 mg) was fractionated by silica gel CC and eluted with CH_2_Cl_2_-acetone (10:1, v/v) to produce seven sub-fractions (C11.1–C11.7). Following a similar procedure to that used for C7.5, sub-fraction C11.4 (140 mg) was subjected to semi-preparative RP-HPLC using MeOH and H_2_O in a 0.1% TFA gradient (50:50–70:30, v/v) at a ﬂow rate of 5 ml/min as a mobile phase to give compounds 1 (5.5 mg) and 4 (5.0 mg).

### Characterization of Potential Resistance Inducers Isolated From B. thuringiensis JCK-1233 Strain Culture

**Figure 3:**
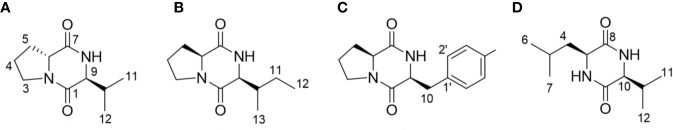
Structural analyses of the isolated diketopiperazines from *Bacillus thuringiensis* JCK-1233. **(A)** *cyclo*-(D-Pro-L-Val), **(B)** *cyclo*-(L-Pro-L-Ile), **(C)** *cyclo*-(L-Pro-L-Phe), and **(D)** *cyclo*-(L-Leu-L-Val).

The presence of the DKP ring system in compounds 1–4 was evident from the characteristic carbon chemical shifts of two amide and carbonyl groups (δ_C_ 166.6–172.6 ppm), and proton chemical shifts of the two methine residues (δ_H_ 3.63–4.45 ppm) (**Supplementary Data S2**) (Jayatilake et al., 1996; Fdhila et al., 2003). The evidence of proline as one of the components of DKP (compounds 1–3) was deduced from the presence of three broad methylene multiplets (δ_H_ 1.25–3.65 ppm) in these compounds. Based on the analysis of 1-dimensional NMR data, COSY correlations and literature values, valine, isoleucine, and phenylalanine were identified as the second amino acid residue in compounds 1–3, respectively (Jayatilake et al., 1996; Campo et al., 2009; Li et al., 2012; Ding et al., 2013; Jiang and Yang, 2013), while in compound 4 it was the combination of leucine and valine (Ding et al., 2013). The configuration of the DKPs was determined by analysis of the NOESY spectrum and comparison with the optical rotations in the literature. In addition, in the NOESY spectrums, the NOE interactions between H-6 and H-9 observed in compounds 2 and 3, but not in compound 1, indicated that these two methine protons have the same orientation in compounds 2 and 3, and a different orientation in compound 1. Based on the above analysis and combined with the positive optical rotation values of compounds 1, and the negative optical rotation values of compounds 2 and 3, the structures of compounds 1–3 were elucidated as *cyclo*-(D-Pro-L-Val) (Jayatilake et al., 1996; Shigemori et al., 1998; Fdhila et al., 2003; Campbell et al., 2009), *cyclo*-(L-Pro-L-Ile) (Jayatilake et al., 1996; Fdhila et al., 2003), and *cyclo*-(L-Pro-L-Phe) (Jayatilake et al., 1996; Fdhila et al., 2003), respectively (**Figure 3**). By a similar analysis, the structure of compound 4 was determined as *cyclo*-(L-Leu-L-Val) (Ding et al., 2013).

*cyclo*-(D-Pro-L-Val) (compound 1). Yellowish oil. [α]$\overset{23}{D}$ +43.8 (*c* 0.1, MeOH). ^1^H NMR (500 MHz, CD_3_OD): δ 4.26 (1H, m, H-7), 3.63 (1H, m, H-9), 3.65, 3.51 (each 1H, m, H-3), 2.37, 1.96 (each 1H, m, H-5), 2.17 (1H, m, H-10), 2.04, 1.91 (each 1H, m, H-4), 1.05 (3H, d, *J* = 7.0, H-11), 1.02 (3H, d, *J* = 7.0, H-12). ^13^C NMR (125 MHz, CD_3_OD): δ 171.5 (C-7), 168.1 (C-1), 64.5 (C-9), 59.8 (C-6), 46.8 (C-3), 34.5 (C-10), 30.4 (C-5), 23.0 (C-4), 19.5 (C-11), 18.5 (C-12). HRESI-MS *m*/*z* 197.1283 [M+H]^+^ (calcd for C_10_H_17_N_2_O_2_, 197.1290).

*cyclo*-(L-Pro-L-Ile) (compound 2). Yellowish oil. [α]$\overset{23}{D}$ −25.5 (*c* 0.14, MeOH). ^1^H NMR (500 MHz, CD_3_OD): δ 4.22 (1H, m, H-6), 4.10 (1H, m, H-9), 3.52-3.59 (2H, m, H-3), 2.34, 1.96 (each 1H, m, H-5), 2.20 (1H, m, H-10), 2.04, 1.95 (each 1H, m, H-4), 1.47, 1.34 (each 1H, m, H-11), 1.10 (3H, d, *J* = 7.0, H-13), 0.96 (3H, t, *J* = 7.5, H-12). ^13^C NMR (125 MHz, CD_3_OD): δ 172.6 (C-7), 167.6 (C-1), 60.0 (C-6), 61.3 (C-9), 46.2 (C-3), 37.1 (C-10), 29.6 (C-5), 25.5 (C-11), 23.3 (C-4), 15.6 (C-13), 12.7 (C-12). HRESI-MS *m*/*z* 211.1443 [M+H]^+^ (calcd for C_11_H_19_N_2_O_2_, 211.1447).

*cyclo*-(L-Pro-L-Phe) (compound 3). Yellowish oil. [α]$\overset{23}{D}$−43.9 (*c* 0.16, MeOH). ^1^H NMR (500 MHz, CD_3_OD): δ 7.32 (2H, m, H-3’, 5’), 7.30 (2H, m, H-2’, 6’), 7.28 (1H, m, H-4’), 4.45 (1H, m, H-6), 4.07 (1H, m, H-9), 3.55, 3.34 (each 1H, m, H-3), 3.18 (2H, t, *J* = 4.5, H-10), 2.11, 1.27 (each 1H, m, H-5), 1.80 (2H, m, H-4). ^13^C NMR (125 MHz, CD_3_OD): δ 171.0 (C-7), 166.9 (C-1), 137.5 (C-1’), 131.1 (C-3’, 5’), 129.5 (C-2’, 6’), 128.1 (C-4’), 60.1 (C-9), 57.4 (C-6), 46.1 (C-3), 38.1 (C-10), 29.4 (C-5), 22.9 (C-4). HRESI-MS *m*/*z* 245.1295 [M+H]^+^ (calcd for C_14_H_17_N_2_O_2_, 245.1290).

*cyclo*-(L-Leu-L-Val) (compound 4). White amorphous powder. [α]$\overset{23}{D}$ −38.1 (*c* 0.15, MeOH). ^1^H NMR (500 MHz, CD_3_OD): δ 3.97 (1H, m, H-3), 3.80 (1H, m, H-10), 2.24 (1H, m, H-11), 1.88 (1H, m, H-5), 1.77, 1.63 (each 1H, m, H-4), 1.08 (3H, d, *J* = 7.0, H-12), 1.00 (3H, d, *J* = 6.5, H-7), 0.98 (3H, d, *J* = 7.0, H-13), 0.97 (3H, d, *J* = 6.5, H-6). ^13^C NMR (125 MHz, CD_3_OD): δ 171.4 (C-8), 169.8 (C-1), 61.6 (C-10), 54.5 (C-3), 46.1 (C-4), 33.8 (C-11), 25.4 (C-5), 23.7 (C-7), 21.9 (C-6), 19.4 (C-12), 17.9 (C-13). HRESI-MS *m*/*z* 213.1611 [M+H]^+^ (calcd for C_11_H_21_N_2_O_2_, 213.1603).

### Effect of Foliar Spray and Trunk Injection of DPKs Produced by B. thuringiensis JCK-1233 Against PWD

**Figure 4:**
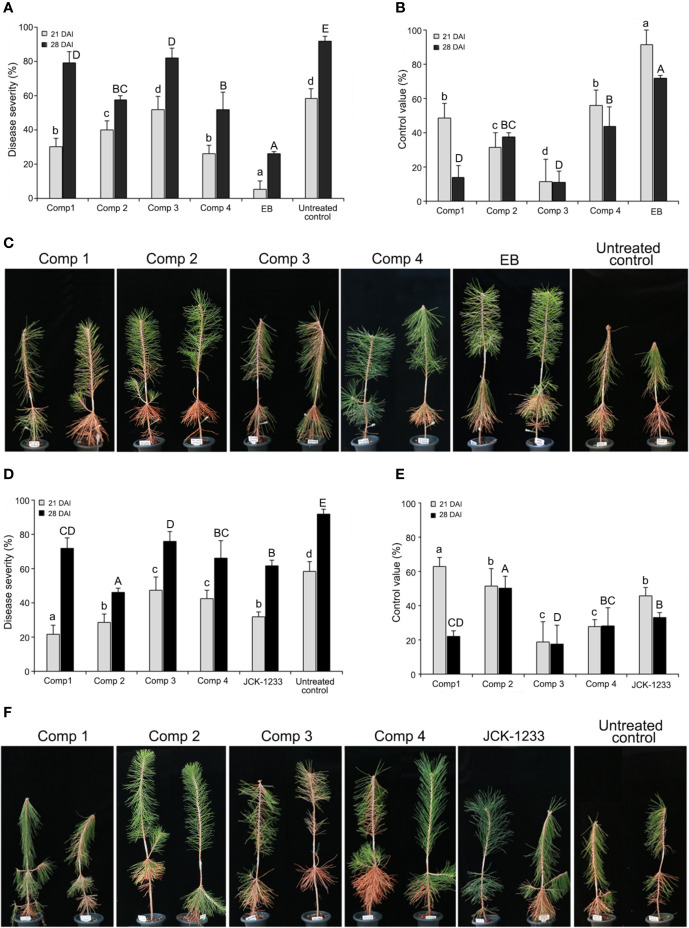
Effect of trunk injection and foliar spray treatments with diketopiperazines on pine wilt disease (PWD). **(A)** Disease severity and **(B)** disease control efficacy of diketopiperazines against PWD at 21 and 28 days after inoculation (DAI) by trunk injection. **(C)** Photographs of the PWD wilting symptoms on pine seedlings treated with diketopiperazines by trunk injection at 28 DAI. **(D)** Disease severity and **(E)** disease control efficacy of diketopiperazines against PWD at 21 and 28 DAI by foliar spray. **(F)** Photographs of the PWD wilting symptoms on pine seedlings treated with diketopiperazines by foliar spray at 28 DAI. Comp 1, *cyclo*-(D-Pro-L-Val); Comp 2, *cyclo*-(L-Pro-L-Ile); Comp 3, *cyclo*-(L-Pro-L-Phe); Comp 4, *cyclo*-(L-Leu-L-Val); EB, emamectin benzoate as a positive control. Error bars represent standard deviation from five replicates. Data was represented as the mean and standard error of two runs with five replicates per run. Different lower and upper case letters shown values that are significantly different (*p <* 0.05) level by Duncan’s test with data at 21 and 28 DAI, respectively.

The effect of trunk injection and foliar spray with four DPKs produced by *B. thuringiensis* JCK-1233, *cyclo*-(D-Pro-L-Val), *cyclo*-(L-Pro-L-Ile), *cyclo*-(L-Pro-L-Phe), and *cyclo*-(L-Leu-L-Val), on PWD control was determined in nematode-inoculated *P. thunbergii* seedlings 21 and 28 days after inoculation. Both trunk injection and foliar spray with a JCK-1233 culture filtrate or the four DPKs showed efficacy in reducing the severity of PWD in nematode-inoculated *P. thunbergii* seedlings (**Figure 4**). For trunk injection, the disease severity after treatment with compounds *cyclo*-(D-Pro-L-Val), *cyclo*-(L-Pro-L-Ile), *cyclo*-(L-Pro-L-Phe), and *cyclo*-(L-Leu-L-Val) were 30.0, 40.0, 51.7, and 25.8%, respectively, after 21 days of inoculation, and progressed to 79.2, 57.5, 81.7, and 51.7%, respectively, after 28 days (**Figure 4A**). The disease control efficacy at 28 days after inoculation was excellent in *cyclo*-(L-Leu-L-Val) and *cyclo*-(L-Pro-L-Ile) (**Figure 4B**).

In foliar spray, disease severity after treatment with *cyclo*-(D-Pro-L-Val), *cyclo*-(L-Pro-L-Ile), *cyclo*-(L-Pro-L-Phe), and *cyclo*-(L-Leu-L-Val) was 21.7, 28.3, 47.3, and 42.2%, respectively, after 21 days of inoculation, and progressed to 71.7, 45.8, 75.8, and 65.8%, respectively, after 28 days (**Figure 4C**). The disease severity and control value trends of the four DPKs upon foliar spray were similar to those observed upon trunk injection, except for *cyclo*-(L-Leu-L-Val) (**Figure 4D**). Specifically, after 28 days of inoculation, the disease control efficacy of *cyclo*-(L-Pro-L-Ile) by foliar spray (50.0%) was the highest among the four compounds, and its disease control efficacy by trunk injection (37.3%) against PWD was also as good as that of *cyclo*-(L-Leu-L-Val) (33.7%), which showed the highest efficacy when trunk injected (**Figures 4C, E****)**.

### Effect of B. thuringiensis JCK-1233 and the Selected Bacterial Active Compound on the Expression of Defense Related Genes In Vivo

**Figure 5:**
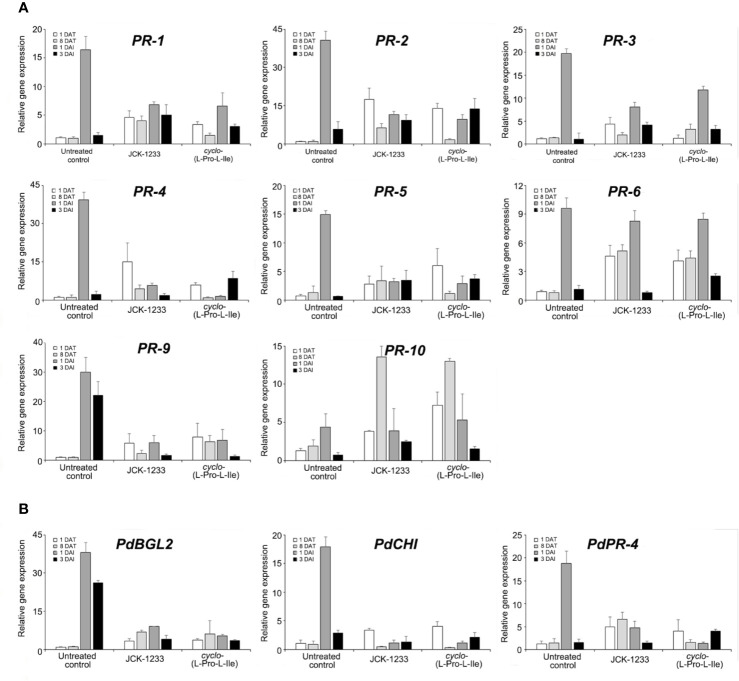
Transcript levels of defense related genes in pine seedlings treated with *Bacillus thuringiensis* JCK-1233 and the bacterial active compound *cyclo*-(L-Pro-L-Ile). The expression of pathogenesis-related (PR) genes amplified with primers oriented from black pine trees (*Pinus thunbergii*) **(A)** and red pine trees (*Pinus densiflora*) **(B)**. Data was presented as the mean and standard error bars of three biological replicates.

The effect of foliar spray with either *B. thuringiensis* JCK-1233 or the bioactive compound *cyclo*-(L-Pro-L-Ile) was tested on the expression of defense-related genes in pine seedlings. The relative expression level of *PR-1* gene was higher at 1 DAT with JCK-1233 and *cyclo*-(L-Pro-L-Ile) treatment (4.64-fold and 3.36-fold increase, respectively) compared to that in untreated control (**Figure 5A**). At 1 DAI with PWN, 6.84-fold and 6.58-fold increases in expression were observed in JCK-1233 and *cyclo*-(L-Pro-L-Ile) treated seedlings, respectively, compared to the untreated control at 1 DAT. However, untreated control seedlings showed a dramatic increase (16.40-fold) in *PR-1* gene expression at 1 DAI with PWN. The relative expression levels of *PR-2*, *PR-3*, *PR-4*, *PR-5*, *PR-9*, *PdBGL2*, and *PdPR-4* also showed a similar pattern to that of *PR-1* (**Figures 5A, B****)**.

On the contrary, the expression of *PR-10* in the untreated control at 1 DAI only increased 4.42-fold compared to that before inoculation, which was similar to the expression after JCK-1233 and *cyclo*-(L-Pro-L-Ile) treatments at the same time point. Moreover, unlike the expression of most pathogenesis-related genes, including *PR-1*, the expression of *PR-10* in JCK-1233 and *cyclo*-(L-Pro-L-Ile) treated plants after 8 days treatment was markedly enhanced (13.57-fold and 13.00-fold, respectively). The expression level of *PR-6* increased markedly at 1 DAI and did not significantly differ between the untreated control (9.63-fold), JCK-1233 treated (8.27-fold), and *cyclo*-(L-Pro-L-Ile) treated (8.49-fold) plants. In addition, after treatment and before nematode inoculation (at 1 DAT and 8 DAT), JCK-1233 and *cyclo*-(L-Pro-L-Ile) treated plants showed a significant increase in the *PR-6* expression level compared to that in untreated plants (**Figure 5A**).

These results suggest that *B. thuringiensis* JCK-1233 and *cyclo*-(L-Pro-L-Ile) enhance the expression of some pathogenesis-related genes in pine plants. However, such enhancement is lower than that produced in nematode-inoculated untreated control plants, except for *PR-6* and *PR-10* genes, indicating that the pathogenesis-related genes evaluated in this study may induce resistance against PWN in a different manner than the general hyposensitive reaction known to occur during SAR.

## Discussion

In this study, *B. thuringiensis* JCK-1233 was selected among 504 isolated bacteria for its ability to induce systemic resistance and suppress the severity of PWD. A DPK *cyclo*-(L-Pro-L-Ile) was identified as a bioactive compound from the selected strain and was shown to induce systemic resistance in pine calli and seedlings. Foliar treatment with the selected strain or the identified compound resulted in a significant reduction in the severity of PWD in inoculated pine seedlings. In general, foliar application using a biocontrol agent or its culture metabolite could represent a less expensive and more applicable approach compared to trunk injection with conventional chemical nematicides in largescale operations and mountainous forests with poor access.

Several DPKs were obtained from *B. thuringiensis* JCK-1233, including *cyclo*-(D-Pro-L-Val), *cyclo*-(L-Pro-L-Ile), *cyclo*-(L-Pro-L-Phe), and *cyclo*-(L-Leu-L-Val). DPKs are among the most common peptide derivatives found in natural products as well as in processed foods and beverages. Of the identified DPKs, foliar application of *cyclo*-(L-Pro-L-Ile) efficiently reduced the incidence of PWD and resulted in the elevated expression of defense-related genes, similar to the effect of *B. thuringiensis* JCK-1233 culture broth. Previous studies have reported the isolation of *cyclo*-(Pro-Ile) from *Aspergillus terreus* (mangrove-associated fungus), *Bacillus pumilus*, *Callyspongia* sp. (marine sponge), and *Trichoderma citrinoviride* (marine-derived fungus) (Shen et al., 2012; Brack et al., 2014; Chen et al., 2014; Zhang et al., 2014). Many studies have reported that DPKs exhibit various effects, including antibacterial, antifungal, antiviral, antitumor, and antitoxin activities (Yan et al., 2004; Noh et al., 2017). Plant growth promoting rhizobacteria (PGPRs) are also known to produce various DPKs that can induce resistance in plants (Noh et al., 2017). Although the plant signaling pathway mediated by DPKs is not clearly characterized yet, *cyclo*-(L-Ala-L-Ile), *cyclo*-(L-Ala-L-Leu), and *cyclo*-(L-Leu-L-Pro) isolated from *Bacillus vallismortis* BS07 elicited disease resistance in *Arabidopsis* against bacterial infection (Noh et al., 2017). However, little is known about the biological functions of *cyclo*-(L-Pro-L-Ile).

It was reported that SAR is associated with the SA signaling pathway, whereas ISR is mediated by the JA and ET signaling pathways (Van Loon et al., 1998). However, they share a lot of similarities both in the result and the mechanisms, and are interconnected by complex signaling networks and crosstalk phenomena (Pieterse et al., 2009; Niu et al., 2016). Several researches have also reported that PGPRs could trigger ISR by concurrently activating the SA- and JA-/ET-signaling pathways, and even activate SA-dependent SAR (De Meyer et al., 1999; Andreasson and Ellis, 2010; Niu et al., 2011; Niu et al., 2016). Song et al. (2017) reported that seed defense biopriming by root-associated *Bacillus gaemokensis* PB69 exhibited combined transcriptional responses with the upregulation of SA, ET, and jasmonic acid signaling. Furthermore, *B. cereus* AR156 is a PGPR that installs ISR to *Pseudomonas syringae* pv. *tomato* in *Arabidopsis* and enhances SAR with increased PR-1 protein expression in plants (Niu et al., 2016). Therefore, we predicted that endophytic bacteria may be able to stimulate the *PR-1* gene expression as well as resistance-inducing activities.

Since the molecular background of induced resistance mechanism in pine trees is not fully understood, we isolated resistance-inducing endophytic bacteria through mass screening using *Arabidopsis* seedlings of the *PR-1pro::GUS* line. Although *PR-1* is the marker gene for *Arabidopsis* SAR and SA-induced defense, it is a good indicator involved in pathogen- or microbe-associated molecular pattern (PAMPs/MAMPs) recognition. The induced resistance mechanism in different plant species was evaluated based on *PR-1* gene expression in *Pinus* calli after treatment with the endophytic bacteria that were selected based on their GUS activity in the *PR-1pro::GUS Arabidopsis* line. Here, we selected *B. thuringiensis* JCK-1233, which stimulated the expression of the *PR-1* gene in *Arabidopsis* and pine calli.

SA-mediated SAR responses are directed against biotrophic pathogens, occurring after the hypersensitive response (HR), which is a highly specific interaction between a plant resistance protein and a pathogenic avirulent, leading to programmed cell death and pathogen growth arrest in the infected plant tissue (Glazebrook, 2005). However, this is literally the case of biotrophic pathogens, such as *Peronospora parasitica*, *Erysiphe* spp., and *Pseudomonas syringae*, not PWNs. Interestingly, the development of PWD caused by PWN *B. xylophilus* has been reported to be closely associated with the HR. HR as a part of the plant immune system is a successful strategy for the control of many potential pests and pathogens, but, in susceptible pine trees against PWNs, this same system causes pine death. Myers (1988) suggested that invasion and early migration of PWNs through tissues enforces a typical HR, such as parenchymal death, toxin production, and leakage of oleoresins and other materials into tracheids. With the rapid migration and propagation of PWNs, the HR spreads throughout the whole plant, and shortly after, susceptible pine trees die. Indeed, several studies in pines have shown a significant increase in the expression of resistance genes to virulent PWNs in susceptible pine trees (Hirao et al., 2012; Lee et al., 2019).

In *P. thunbergii*, the expression of *PR-1*, *PR-2*, *PR-3*, *PR-4*, *PR-5*, and *PR-6* was increased in susceptible trees but not in resistant trees after inoculation with PWNs (Hirao et al., 2012). In *Pinus densiflora*, inoculation of PWN also increased the expression of genes involved in the defense response, such as PR proteins (Lee et al., 2019). Consistent with these results, we observed a marked rise in the expression levels of *PR-1*, *PR-2*, *PR-3*, *PR-4*, *PR-5*, *PR-9*, *PdBGL*, *PdCHI*, and *PdPR-4* in the untreated control compared to JCK-1233 or *cyclo*-(L-Pro-L-Ile) treatment after inoculation with PWNs. In our gene expression analysis using the susceptible species *Pinus thunbergii*, we observed that the multitude of PR genes were upregulated up to 15–41-fold at 1 day after infection with PWNs compared to before infection, indicating that the HR can occur rapidly in pine seedlings infected with PWNs. Importantly, the untreated control group developed an HR much faster than the *B. thuringiensis* JCK-1233 and *cyclo*-(L-Pro-L-Ile) treated groups. Therefore, if there are treatments that can inhibit the migration of PWNs and alleviate the HR during infection, they may be helpful to control PWD. Our results suggest that disease resistance in pine trees may be caused by a moderate hypersensitive reaction.

Fitness-defense balance is important in terms of plant resistance against pathogens (Hirao et al., 2012). It is reasonable to think that plants express their inducible defense only if the protection against pathogens outweighs the costs of the resistance. However, in susceptible pine trees, plants develop an intense HR against PWNs. Although susceptible pine trees are unable to overcome infection, they develop excessive resistance systems, losing their fitness-defense balance and eventually dying. Pine seedlings treated with either *B. thuringiensis* JCK-1233 or its active compound DPK *cyclo*-(L-Pro-L-Ile) moderately increased expression of PR genes compared to that of the untreated control before and after inoculation with PWNs, suggesting that a moderate hypersensitive reaction can be a factor in their resistance against PWD. Although the exact mechanism behind the resistance induced by JCK-1233 and its active compound, DPK *cyclo*-(L-Pro-L-Ile), was not investigated, we hypothesize that it is involved in maintaining the fitness-defense balance. In addition, when PWNs infect susceptible pine trees, JCK-1233 and its active compound DPK *cyclo*-(L-Pro-L-Ile) may elicit a resistance consisting of interconnected complex signaling networks and, consequently, result in a moderate hypersensitive reaction. Therefore, we emphasize the importance of future investigations using molecular biological analyses to determine the functional mechanisms involved in the moderate HR induced by endophytic bacteria or DPKs, especially in susceptible pine trees, such as *P. thunbergii*, *P. koraiensis*, *P. densiflora*, and *P. pinaster*.

Among the tested PR genes, the expression patterns of *PR-6* and *PR-10* were different from those of other genes related to the HR, which were markedly expressed when susceptible pine trees were infected with PWNs. After inoculation with PWNs, there was no significant difference in the expression of *PR-6* and *PR-10* between the untreated and treated groups (**Figure 5**). Moreover, *B. thuringiensis* JCK-1233 and *cyclo*-(L-Pro-L-Ile) treated pine seedlings exhibited significantly higher expression levels of *PR-6* and *PR-10* than that in the untreated control before inoculation with PWN. PR-6 is known to be active in nematodes and insects, acting as a proteinase inhibitor (Devi et al., 2017). In plants, induced proteinase inhibitors often have putative proteinases targeted to the digestive tract of specific insect predators (Heitz et al., 1999). Thus, a protein fraction from soybean inhibited growth and proteolytic activity of the meal worm *Tribolium confusum in vitro* (Lipke et al., 1954). PR-10 was shown to be a ribonuclease-like protein acting against a digestive proteinase secreted by the root knot nematode *Meloidogyne incognita*, which results in a nematostatic condition *in vitro* (Andrade et al., 2010). Along the same lines, PR-10 is predicted to act as a proteinase against cellulases, β-1,3-glucanase, and pectate lyases secreted by PWNs (Kikuchi et al., 2004; Kikuchi et al., 2005; Kikuchi et al., 2006; Hirao et al., 2012). Although both PR-6 and PR-10 are not potent nematicidal proteins, they might have a role in the suppression of PWN propagation and migration during the early infection stage, representing an element in the induced resistance theory, moderate HR, proposed in this study.

In summary, *B. thuringiensis* JCK-1233 was selected among 504 isolated bacterial strains for its possible pine systemic resistance-inducing activity against PWD. Although the selected *B. thuringiensis* JCK-1233 did not have a direct nematicidal effect, foliar treatment of pine seedlings resulted in a significant reduction in PWD severity to a level comparable to that of EB trunk injection. In addition, out of the four DPKs isolated from the selected strain, the activity of *cyclo*-(L-Pro-L-Ile) was considered to be a main factor involved in the induction of pine seedling resistance by *B. thuringiensis* JCK-1233. Foliar application with *cyclo*-(L-Pro-L-Ile) showed better control efficacy compared to trunk injection, as observed at 28 DAI with PWN. Foliar application has two major advantages; it can effectively control PWD at a low cost, and it can be applied in the management of PWD in forests or areas that are inaccessible to humans. Treatment with *B. thuringiensis* JCK-1233 or the bioactive compound, the DPK *cyclo*-(L-Pro-L-Ile), moderately enhanced the expression of various pathogenesis-related genes associated with plant immunity. As a result, a rapid and intense HR was suppressed, and a fitness-defense balance was adequately maintained. Based on our results, it may be possible to develop an eco-friendly agent for the control of PWD utilizing our proposed agent as an aerial application. This study could be the cornerstone for prospective studies on the induced resistance against PWD in susceptible pine trees worldwide.

## Data Availability Statement

The original contributions presented in the study are included in the article/**Supplementary Material**; further inquiries can be directed to the corresponding authors.

## Author Contributions

AP, S-IJ, Y-SS, and J-CK conceived this study. AP, S-IJ, HJ, JuK, NK, MH, MM, JunK, CL, BM, Y-SS, and J-CK performed the experiments. AP, S-IJ, CL, MM, BM, Y-SS, and J-CK analyzed the data. AP, S-IJ, MH, BM, Y-SS, and J-CK wrote the manuscript. All authors contributed to the article and approved the submitted version.

## Funding

This research was supported by the National Institute of Forest Science, South Korea (FE0702-2016-11-2020).

## Conflict of Interest

The authors declare that the research was conducted in the absence of any commercial or financial relationships that could be construed as a potential conflict of interest.
