# Supplementary material for: A Diketopiperazine, Cyclo-(L-Pro-L-Ile), Derived From Bacillus thuringiensis JCK-1233 Controls Pine Wilt Disease by Elicitation of Moderate Hypersensitive Reaction
Source: Front Plant Sci. 2020 Jul 8;11:1023. doi: 10.3389/fpls.2020.01023 (PMC7396504; doi:10.3389/fpls.2020.01023)
Supplement: Supplementary file 1 [file DataSheet_1.docx]

**Supplementary Data S1**

**A Diketopiperazine, *Cyclo*-(L-Pro-L-Ile), Derived from *Bacillus thuringiensis* JCK-1233 Controls Pine Wilt Disease by Elicitation of Moderate Hypersensitive Reaction**

**Ae Ran Park^1†^, Se-In Jeong^1†^, Hee Won Jeon^1^, Jueun Kim ^2^, Namgyu Kim^3^, Manh Tuan Ha^4^, Mohamed Mannaa^3^, Junheon Kim^5^, Chul Won Lee ^2^, Byung Sun Min^4^, Young-Su Seo^3*^, and Jin-Cheol Kim^1*^**

^1^Department of Agricultural Chemistry, Institute of Environmentally Friendly Agriculture, College of Agriculture and Life Sciences, Chonnam National University, Gwangju, South Korea

^2^Department of Chemistry, Chonnam National University, Gwangju, South Korea

^3^Department of Integrated Biological Science, College of Natural Science, Pusan National University, Busan, South Korea

^4^College of Pharmacy, Drug Research and Development Center, Daegu Catholic University, Gyeongbuk, South Korea.

^5^Forest Insect Pests and Diseases Division, National Institute of Forest Science, Seoul, South Korea

**^*^**Correspondence:

Jin-Cheol Kim, [kjinc@jnu.ac.kr](mailto:kjinc@jnu.ac.kr); Young-Su Seo, yseo2011@pusan.ac.kr

**^†^**These authors have contributed equally to this work.

Running title: Diketopiperazine-induced resistance in pine

**Supplementary Data S1.** Effects of endophytic bacterial strains on GUS expression induced by explanting in leaf tissue of AtPR1-GUS *Arabidopsis* plant seedlings

| Strain | GUS activity | Strain | GUS activity | Strain | GUS activity |
| --- | --- | --- | --- | --- | --- |
| JCK-726 | - | JCK-1062 | - | JCK-1230 | - |
| JCK-727 | - | JCK-1063 | - | JCK-1231 | - |
| JCK-736 | - | JCK-1064 | - | JCK-1232 | - |
| JCK-737 | - | JCK-1065 | - | JCK-1233 | * |
| JCK-738 | - | JCK-1066 | - | JCK-1234 | - |
| JCK-742 | - | JCK-1067 | - | JCK-1235 | - |
| JCK-743 | - | JCK-1068 | - | JCK-1236 | - |
| JCK-744 | - | JCK-1069 | - | JCK-1237 | - |
| JCK-745 | - | JCK-1070 | - | JCK-1238 | - |
| JCK-748 | - | JCK-1071 | - | JCK-1239 | - |
| JCK-749 | - | JCK-1072 | - | JCK-1240 | - |
| JCK-750 | - | JCK-1073 | - | JCK-1241 | - |
| JCK-751 | - | JCK-1074 | - | JCK-1242 | - |
| JCK-752 | - | JCK-1075 | - | JCK-1243 | - |
| JCK-753 | * | JCK-1076 | - | JCK-1244 | - |
| JCK-754 | - | JCK-1077 | - | JCK-1245 | - |
| JCK-755 | - | JCK-1078 | - | JCK-1246 | - |
| JCK-756 | - | JCK-1079 | - | JCK-1247 | - |
| JCK-757 | - | JCK-1080 | - | JCK-1248 | - |
| JCK-758-1 | * | JCK-1081 | - | JCK-1249 | - |
| JCK-758-2 | * | JCK-1082 | - | JCK-1250 | - |
| JCK-759 | - | JCK-1083 | - | JCK-1251 | - |
| JCK-761 | * | JCK-1084 | - | JCK-1252 | - |
| JCK-762 | - | JCK-1085 | - | JCK-1253 | - |
| JCK-763 | - | JCK-1086 | - | JCK-1254 | - |
| JCK-764 | - | JCK-1087 | - | JCK-1255 | - |
| JCK-765 | - | JCK-1088 | - | JCK-1256 | - |
| JCK-766 | - | JCK-1089 | - | JCK-1257 | - |
| JCK-767 | * | JCK-1090 | - | JCK-1258 | - |
| JCK-768 | - | JCK-1091 | - | JCK-1259 | - |
| JCK-769 | - | JCK-1092 | - | JCK-1260 | - |
| JCK-770 | - | JCK-1093 | - | JCK-1261 | - |
| JCK-771 | - | JCK-1094 | - | JCK-1262 | - |
| JCK-772 | - | JCK-1095 | - | JCK-1263 | - |
| JCK-773 | - | JCK-1096 | - | JCK-1264 | - |
| JCK-774 | - | JCK-1097 | - | JCK-1265 | - |
| JCK-775 | - | JCK-1098 | - | JCK-1266 | * |
| JCK-776 | - | JCK-1099 | - | JCK-1267 | - |
| JCK-777 | - | JCK-1100 | - | JCK-1268 | - |
| JCK-778 | - | JCK-1101 | - | JCK-1269 | - |
| JCK-779 | - | JCK-1102 | - | JCK-1270 | - |
| JCK-780 | - | JCK-1103 | - | JCK-1271 | - |
| JCK-781 | - | JCK-1104 | - | JCK-1272 | - |
| JCK-782 | - | JCK-1105 | - | JCK-1273 | - |
| JCK-783 | - | JCK-1106 | - | JCK-1274 | - |
| JCK-925 | - | JCK-1107 | - | JCK-1275 | - |
| JCK-926 | - | JCK-1108 | - | JCK-1276 | - |
| JCK-927 | - | JCK-1109 | - | JCK-1277 | - |
| JCK-928 | - | JCK-1110 | - | JCK-1278 | - |
| JCK-929 | - | JCK-1111 | - | JCK-1279 | - |
| JCK-930 | - | JCK-1112 | - | JCK-1280 | - |
| JCK-931 | - | JCK-1113 | - | JCK-1281 | - |
| JCK-932 | - | JCK-1114 | - | JCK-1282 | - |
| JCK-933 | - | JCK-1115 | - | JCK-1283 | - |
| JCK-934 | - | JCK-1116 | - | JCK-1284 | - |
| JCK-935 | - | JCK-1117 | - | JCK-1285 | - |
| JCK-936 | - | JCK-1118 | - | JCK-1286 | - |
| JCK-937 | - | JCK-1119 | - | JCK-1287 | * |
| JCK-938 | - | JCK-1120 | - | JCK-1288 | * |
| JCK-939 | - | JCK-1121 | - | JCK-1289 | - |
| JCK-940 | - | JCK-1122 | - | JCK-1290 | - |
| JCK-941 | - | JCK-1123 | - | JCK-1291 | - |
| JCK-942 | - | JCK-1124 | - | JCK-1292 | - |
| JCK-943 | - | JCK-1125 | - | JCK-1293 | - |
| JCK-944 | - | JCK-1126 | - | JCK-1294 | - |
| JCK-945 | - | JCK-1127 | - | JCK-1295 | - |
| JCK-946 | - | JCK-1128 | - | JCK-1296 | - |
| JCK-947 | * | JCK-1129 | - | JCK-1297 | - |
| JCK-949 | - | JCK-1130 | - | JCK-1298 | - |
| JCK-950 | - | JCK-1131 | - | JCK-1299 | - |
| JCK-951 | - | JCK-1132 | - | JCK-1300 | - |
| JCK-952 | - | JCK-1133 | - | JCK-1301 | - |
| JCK-953 | - | JCK-1134 | - | JCK-1302 | - |
| JCK-954 | - | JCK-1135 | - | JCK-1303 | - |
| JCK-955 | - | JCK-1136 | - | JCK-1304 | - |
| JCK-956 | - | JCK-1137 | - | JCK-1305 | - |
| JCK-957 | - | JCK-1138 | - | JCK-1306 | - |
| JCK-958 | - | JCK-1139 | - | JCK-1307 | * |
| JCK-959 | - | JCK-1140 | - | JCK-1308 | * |
| JCK-960 | - | JCK-1141 | - | JCK-1309 | * |
| JCK-961 | - | JCK-1142 | - | JCK-1310 | - |
| JCK-962 | - | JCK-1143 | - | JCK-1311 | - |
| JCK-963 | - | JCK-1144 | - | JCK-1312 | - |
| JCK-964 | - | JCK-1145 | - | JCK-1313 | - |
| JCK-965 | - | JCK-1146 | - | JCK-1314 | - |
| JCK-966 | - | JCK-1147 | - | JCK-1315 | - |
| JCK-967 | - | JCK-1148 | - | JCK-1316 | - |
| JCK-968 | - | JCK-1149 | - | JCK-1317 | - |
| JCK-969 | - | JCK-1150 | - | JCK-1318 | * |
| JCK-970 | - | JCK-1151 | - | JCK-1319 | - |
| JCK-971 | - | JCK-1152 | - | JCK-1320 | * |
| JCK-972 | - | JCK-1153 | - | JCK-1321 | - |
| JCK-973 | - | JCK-1154 | - | JCK-1322 | - |
| JCK-974 | - | JCK-1155 | - | JCK-1323 | - |
| JCK-975 | - | JCK-1156 | - | JCK-1324 | - |
| JCK-976 | - | JCK-1157 | - | JCK-1325 | - |
| JCK-977 | - | JCK-1158 | - | JCK-1326 | - |
| JCK-978 | - | JCK-1159 | - | JCK-1327 | - |
| JCK-979 | - | JCK-1160 | - | JCK-1328 | * |
| JCK-980 | - | JCK-1161 | - | JCK-1329 | - |
| JCK-981 | - | JCK-1162 | - | JCK-1330 | - |
| JCK-982 | - | JCK-1163 | - | JCK-1331 | - |
| JCK-983 | - | JCK-1164 | - | JCK-1332 | - |
| JCK-984 | - | JCK-1165 | - | JCK-1333 | * |
| JCK-985 | - | JCK-1166 | - | JCK-1334 | - |
| JCK-986 | - | JCK-1167 | - | JCK-1335 | - |
| JCK-987 | - | JCK-1168 | - | JCK-1336 | - |
| JCK-988 | - | JCK-1169 | - | JCK-1337 | - |
| JCK-1000 | - | JCK-1170 | - | JCK-1338 | - |
| JCK-1001 | - | JCK-1171 | - | JCK-1339 | - |
| JCK-1002 | - | JCK-1172 | - | JCK-1340 | - |
| JCK-1003 | - | JCK-1173 | - | JCK-1341 | - |
| JCK-1004 | - | JCK-1174 | - | JCK-1342 | - |
| JCK-1005 | * | JCK-1175 | - | JCK-1343 | - |
| JCK-1006 | - | JCK-1176 | - | JCK-1344 | - |
| JCK-1007 | - | JCK-1177 | - | JCK-1345 | - |
| JCK-1008 | - | JCK-1178 | - | JCK-1346 | - |
| JCK-1009 | - | JCK-1179 | - | JCK-1347 | - |
| JCK-1010 | - | JCK-1180 | * | JCK-1348 | - |
| JCK-1011 | - | JCK-1181 | - | JCK-1349 | - |
| JCK-1012 | - | JCK-1182 | * | JCK-1350 | - |
| JCK-1013 | - | JCK-1183 | - | JCK-1351 | - |
| JCK-1014 | - | JCK-1184 | - | JCK-1352 | - |
| JCK-1015 | - | JCK-1185 | - | JCK-1353 | - |
| JCK-1016 | - | JCK-1186 | - | JCK-1354 | - |
| JCK-1017 | - | JCK-1187 | * | JCK-1355 | - |
| JCK-1018 | - | JCK-1188 | - | JCK-1356 | - |
| JCK-1019 | - | JCK-1189 | - | JCK-1357 | - |
| JCK-1020 | - | JCK-1190 | - | JCK-1358 | - |
| JCK-1021 | - | JCK-1191 | - | JCK-1359 | - |
| JCK-1022 | - | JCK-1192 | - | JCK-1360 | - |
| JCK-1023 | - | JCK-1193 | - | JCK-1361 | - |
| JCK-1024 | - | JCK-1194 | - | JCK-1362 | - |
| JCK-1025 | - | JCK-1195 | - | JCK-1363 | - |
| JCK-1026 | - | JCK-1196 | - | JCK-1364 | - |
| JCK-1027 | - | JCK-1197 | - | JCK-1365 | - |
| JCK-1028 | - | JCK-1198 | - | JCK-1366 | - |
| JCK-1029 | - | JCK-1199 | - | JCK-1367 | - |
| JCK-1032 | - | JCK-1200 | - | JCK-1368 | - |
| JCK-1033 | - | JCK-1201 | - | JCK-1369 | - |
| JCK-1034 | - | JCK-1202 | - | JCK-1370 | - |
| JCK-1035 | - | JCK-1203 | - | JCK-1371 | - |
| JCK-1036 | - | JCK-1204 | - | JCK-1372 | - |
| JCK-1037 | - | JCK-1205 | - | JCK-1373 | - |
| JCK-1038 | - | JCK-1206 | - | JCK-1374 | - |
| JCK-1039 | - | JCK-1207 | - | JCK-1375 | - |
| JCK-1040 | - | JCK-1208 | - | JCK-1376 | - |
| JCK-1041 | - | JCK-1209 | - | JCK-1377 | - |
| JCK-1042 | - | JCK-1210 | - | JCK-1378 | - |
| JCK-1043 | - | JCK-1211 | - | JCK-1379 | - |
| JCK-1044 | - | JCK-1212 | - | JCK-1380 | - |
| JCK-1045 | - | JCK-1213 | - | JCK-1381 | - |
| JCK-1046 | - | JCK-1214 | - | JCK-1382 | - |
| JCK-1047 | - | JCK-1215 | - | JCK-1383 | - |
| JCK-1048 | - | JCK-1216 | - | JCK-1384 | - |
| JCK-1049 | - | JCK-1217 | * | JCK-1385 | - |
| JCK-1050 | - | JCK-1218 | - | JCK-1386 | - |
| JCK-1051 | - | JCK-1219 | - | JCK-1387 | - |
| JCK-1052 | - | JCK-1220 | - | JCK-1388 | - |
| JCK-1053 | - | JCK-1221 | - | JCK-1389 | - |
| JCK-1056 | - | JCK-1222 | * | JCK-1390 | - |
| JCK-1055 | - | JCK-1223 | - | JCK-1391 | - |
| JCK-1056 | - | JCK-1224 | - | JCK-1392 | - |
| JCK-1057 | - | JCK-1225 | - | JCK-1393 | - |
| JCK-1058 | - | JCK-1226 | - | JCK-1394 | - |
| JCK-1059 | - | JCK-1227 | - | JCK-1395 | - |
| JCK-1060  JCK-1061 | -  - | JCK-1228  JCK-1229 | -  * | JCK-1396  JCK-1397 | -  - |

-, no visible GUS expression; *, induced GUS expression, All data were obtained from three replicates.
