# Supplementary material for: A Diketopiperazine, Cyclo-(L-Pro-L-Ile), Derived From Bacillus thuringiensis JCK-1233 Controls Pine Wilt Disease by Elicitation of Moderate Hypersensitive Reaction
Source: Front Plant Sci. 2020 Jul 8;11:1023. doi: 10.3389/fpls.2020.01023 (PMC7396504; doi:10.3389/fpls.2020.01023)
Supplement: Supplementary file 2 [file DataSheet_2.docx]

**Supplementary Data S2**

**A Diketopiperazine, *Cyclo*-(L-Pro-L-Ile), Derived from *Bacillus thuringiensis* JCK-1233 Controls Pine Wilt Disease by Elicitation of Moderate Hypersensitive Reaction**

**Ae Ran Park^1†^, Se-In Jeong^1†^, Hee Won Jeon^1^, Jueun Kim ^2^, Namgyu Kim^3^, Manh Tuan Ha^4^, Mohamed Mannaa^3^, Junheon Kim^5^, Chul Won Lee ^2^, Byung Sun Min^4^, Young-Su Seo^3*^, and Jin-Cheol Kim^1*^**

^1^Department of Agricultural Chemistry, Institute of Environmentally Friendly Agriculture, College of Agriculture and Life Sciences, Chonnam National University, Gwangju, South Korea

^2^Department of Chemistry, Chonnam National University, Gwangju, South Korea

^3^Department of Integrated Biological Science, College of Natural Science, Pusan National University, Busan, South Korea

^4^College of Pharmacy, Drug Research and Development Center, Daegu Catholic University, Gyeongbuk, South Korea.

^5^Forest Insect Pests and Diseases Division, National Institute of Forest Science, Seoul, South Korea

**^*^**Correspondence:

Jin-Cheol Kim, [kjinc@jnu.ac.kr](mailto:kjinc@jnu.ac.kr); Young-Su Seo, yseo2011@pusan.ac.kr

**^†^**These authors have contributed equally to this work.

Running title: Diketopiperazine-induced resistance in pine

Figure S1.1. ^1^H NMR spectrum (500 MHz, CD_3_OD) of compound 1 (*cyclo*-(D-Pro-L-Val))

Figure S1.2. ^13^C NMR spectrum (125 MHz, CD_3_OD) of compound 1 (*cyclo*-(D-Pro-L-Val))

Figure S1.3. DEPT spectrum (125 MHz, CD_3_OD) of compound 1 (*cyclo*-(D-Pro-L-Val))

Figure S1.4. HMQC spectrum of compound 1 (*cyclo*-(D-Pro-L-Val))

Figure S1.5. ^1^H-^1^H COSY spectrum of compound 1 (*cyclo*-(D-Pro-L-Val))

Figure S1.6. HMBC spectrum of compound 1 (*cyclo*-(D-Pro-L-Val))

Figure S1.7. ^1^H-^1^H NOESY spectrum of compound 1 (*cyclo*-(D-Pro-L-Val))

Figure S1.8. HRESIMS spectrum of compound 1 (*cyclo*-(D-Pro-L-Val))

Figure S2.1. ^1^H NMR spectrum (500 MHz, CD_3_OD) of compound 2 (*cyclo*-(L-Pro-L-Ile))

Figure S2.2. ^13^C NMR spectrum (125 MHz, CD_3_OD) of compound 2 (*cyclo*-(L-Pro-L-Ile))

Figure S2.3. DEPT spectrum (125 MHz, CD_3_OD) of compound 2 (*cyclo*-(L-Pro-L-Ile))

Figure S2.4. HMQC spectrum of compound 2 (*cyclo*-(L-Pro-L-Ile))

Figure S2.5. ^1^H-^1^H COSY spectrum of compound 2 (*cyclo*-(L-Pro-L-Ile))

Figure S2.6. HMBC spectrum of compound 2 (*cyclo*-(L-Pro-L-Ile))

Figure S2.7. ^1^H-^1^H NOESY spectrum of compound 2 (*cyclo*-(L-Pro-L-Ile))

Figure S2.8. HRESIMS spectrum of compound 2 (*cyclo*-(L-Pro-L-Ile))

Figure S3.1. ^1^H NMR spectrum (500 MHz, CD_3_OD) of compound 3 (*cyclo*-(L-Pro-L-Phe))

Figure S3.2. ^13^C NMR spectrum (125 MHz, CD_3_OD) of compound 3 (*cyclo*-(L-Pro-L-Phe))

Figure S3.3. DEPT spectrum (125 MHz, CD_3_OD) of compound 3 (*cyclo*-(L-Pro-L-Phe))

Figure S3.4. HMQC spectrum of compound 3 (*cyclo*-(L-Pro-L-Phe))

Figure S3.5. ^1^H-^1^H COSY spectrum of compound 3 (*cyclo*-(L-Pro-L-Phe))

Figure S3.6. HMBC spectrum of compound 3 (*cyclo*-(L-Pro-L-Phe))

Figure S3.7. ^1^H-^1^H NOESY spectrum of compound 3 (*cyclo*-(L-Pro-L-Phe))

Figure S3.8. HRESIMS spectrum of compound 3 (*cyclo*-(L-Pro-L-Phe))

Figure S4.1. ^1^H NMR spectrum (500 MHz, CD_3_OD) of compound 4 (*cyclo*-(L-Leu-L-Val))

Figure S4.2. ^13^C NMR spectrum (125 MHz, CD_3_OD) of compound 4 (*cyclo*-( L-Leu-L-Val))

Figure S4.3. DEPT spectrum (125 MHz, CD_3_OD) of compound 4 (*cyclo*-(L-Leu-L-Val))

Figure S4.4. HMQC spectrum of compound 4 (*cyclo*-(L-Leu-L-Val))

Figure S4.5. ^1^H-^1^H COSY spectrum of compound 4 (*cyclo*-(L-Leu-L-Val))

Figure S4.6. HMBC spectrum of compound 4 (*cyclo*-(L-Leu-L-Val))

Figure S4.7. ^1^H-^1^H NOESY spectrum of compound 4 (*cyclo*-(L-Leu-L-Val))

Figure S4.8. HRESIMS spectrum of compound 4 (*cyclo*-(L-Leu-L-Val))
